# Patient-caregiver communication concordance in cancer—refinement of the Cancer Communication Assessment Tool in an Australian sample

**DOI:** 10.1007/s00520-022-07163-7

**Published:** 2022-05-25

**Authors:** Natasha Michael, Alex Gorelik, Ekavi Georgousopoulou, Merlina Sulistio, Patrick Tee, Katherine Hauser, David Kissane

**Affiliations:** 1 Supportive, Psychosocial and Palliative Care Research Department, Cabrini Health, 646 High Street, Prahran, Melbourne, VIC Australia; 2 School of Medicine, University of Notre Dame Australia , NSW, Australia; 3 Faculty of Medicine, Nursing and Health Sciences, Monash University, Melbourne VIC, Australia; 4 Monash-Cabrini Department of Musculoskeletal Health and Clinical Epidemiology, Cabrini Health, Melbourne, VIC Australia; 5 Department of Epidemiology and Preventive Medicine, School of Public Health and Preventive Medicine, Monash University, Melbourne VIC, Australia; 6 Department of Medicine (RMH), University of Melbourne, Melbourne VIC, Australia; 7 Sacred Heart Health Service, St. Vincent’s Hospital, Sydney, NSW Australia

**Keywords:** Cancer, Communication, Caregiver, Validation, Questionnaire

## Abstract

**Purpose:**

The objective of this study was to expand the international psychometric validation of the Cancer Communication Assessment Tool for Patients and Families (CCAT-PF) within a sample of Australian cancer patients.

**Methods:**

Survey data from 181 cancer patient-caregiver dyads ≥ 18 years of age with solid or haematological cancers were analysed (85.4% response rate). Spearman’s rho was used to examine the correlation between CCAT-P and CCAT-F scores and weighted kappa the agreement between them. Exploratory factor analysis using scree plot and Kaiser-Guttman criteria was conducted to evaluate the scale structure. Cronbach’s *α* and Pearson correlation coefficients were used to measure internal consistency and concurrent validity respectively.

**Results:**

Mean scores were the following: CCAT-P 46.2 (9.8), CCAT-F 45.7 (9.4), and CCAT-PF 24.1 (8.0). We confirmed the poor concordance between patient and caregiver reporting of items in the CCAT-PF, with all but two items having weighted kappa values < 0.20 and Spearman’s rho < 0.19. We derived a three-factor solution, *disclosure*, *limitation of treatment*, and *treatment decision making*, with reliability ranging from Cronbach’s *α* = 0.43–0.53. The CCAT-P and CCAT-F showed strong correlations with preparation for decision-making (CCAT-P: *r* = 0.0.92; CCATF: *r* = 0.0.93) but were weakly associated with patient/caregiver distress related with having difficult conversations on future care planning.

**Conclusion:**

Preliminary validation of the CCAT-PF in the Australian setting has shown some similar psychometric properties to previously published studies, further supporting its potential utility as a tool to assess patient-caregiver dyadic communication.

**Trial registration:**

ACTRN12620001035910 12/10/2020 retrospectively registered.

**Supplementary Information:**

The online version contains supplementary material available at 10.1007/s00520-022-07163-7.

## Background

A diagnosis of cancer is disruptive to family functioning, eliciting anxiety, distress, and not uncommonly causing interpersonal conflict between family members [1, 2]. Communication in cancer care is thus crucial but is recognised as complex, requiring a multifaceted approach in ensuring appropriate content, affect and delivery across multiple settings [3, 4]. Such complexity brings about discordance in patient-caregiver communication which is well recognised [5] and arises out of challenges in acquiring information about diagnosis, prognosis and treatment options [6], poor patient-caregiver goal alignment [7], limited supports to enable optimal coping [1], and an overall avoidance of open communication [8].

Cancer caregivers are thus now routinely recognised as forming part of the triad of care with patients and health professionals, with the goal of building trusting relationships, sharing information, eliciting concerns, and enabling patients and families to talk about their feelings and concerns [9, 10]. Patient-caregiver dyadic communication in cancer is to be encouraged, but has been described as ‘work’ [11] and is consequential, with poor communication increasing patient and caregiver depression, anxiety [12], and relational satisfaction [1]. Conversely, proactive discourse improves dyadic coping [1] and resilience [13], allowing for recovery from the many stresses associated with a cancer diagnosis.

Whilst methods and instruments exist to assess individual patient [3, 14] and caregiver communication [15, 16], there remain few valid and reliable instruments designed to capture the level of agreement (concordance) or disagreement (discordance) in cancer patient-caregiver dyadic communication to assist targeted interventions. One such instrument, the Cancer Communication Assessment Tool for Patients and Families (CCAT-PF) [17], was designed to assess patient-caregiver congruence in communication, with higher scores signifying greater discord.

Preliminary research on the psychometrics of the CCAT-PF by Sminoff et al. in 190 American lung cancer patient-caregiver dyads demonstrated a mean CCAT-PF of 26.9 (*SD* 8.8), with test–retest reliability of 0.35 and Cronbach’s *α* of 0.49 [17]. Likewise, a Korean study of 990 heterogeneous cancer dyads identified a slightly lower mean CCAT-PF (23.7), with moderate internal consistency (Cronbach’s *α*: CCAT-P = 0.52, CCAT-F = 0.50, CCAT-PF = 0.60) [18]. This is in keeping with the CCAT-PF not being the summed scale of a single construct but of eight independent constructs that do not correlate well with each other. A subsequent German cross-sectional study of 189 cancer patient-caregiver dyads completed an exploratory factor analysis and described four factors within the CCAT-PF: disclosure (Cronbach’s *α* = 0.66), limitation of treatment (Cronbach’s *α* = 0.51), family involvement in treatment decisions (Cronbach’s *α* = 0.68), and continuing treatment (Cronbach *α* = 0.51) [19]. The disclosure subscale was found to be a valid and reliable instrument for identifying conflicting communication in at-risk patient-caregiver dyads, correlating with patient distress (*r* = 0.30, *p* < 0.0001), specific unmet needs (*r* = 0.25–0.32, *p* < 0.001), and negatively with social/family well-being (*r* =  − 0.31, *p* < 0.0001) [19].

High conflict scores on the CCAT-PF significantly correlated with greater patient depression and lower family expression of feelings and cohesion [17] but weakly with patient/caregiver perceived family avoidance of cancer care [18]. Both the CCAT-P and CCAT-F scores were weakly associated with mental health and quality of life outcomes [18]. The CCAT-PF has also been used longitudinally, with a recent 2-year study of 171 haematological cancer patient-caregiver dyads demonstrating that communication is dynamic over time, with race, income, and the quality of dyadic relationships affecting patterns of concordance [20].

This exploratory study was undertaken to examine the potential applicability of the CCAT-PF within the Australian setting. We sought to explore its early psychometric properties (internal consistency) and exploratory factor analysis across heterogeneous tumour types.

## Methods

### Study design and sample

This study formed part of a randomised control trial (RCT) exploring advance care planning (ACP) in cancer patient-caregiver dyads [21, 22], which follows through from preliminary published studies [23–26]. Dyads were randomised to a video supported intervention demonstrating conversations on end of life values or usual care. The study was conducted at an 850-bed metropolitan teaching hospital in Melbourne, Australia. Patients diagnosed with solid or haematological cancer were recruited from the oncology and palliative care services and were asked to nominate a willing caregiver. Patients and caregivers who were insufficiently proficient in English, aged > 18, or unable to consent due to cognitive barriers were excluded.

Following enrolment and written consent and prior to randomisation, patient-caregiver dyads completed an anonymous questionnaire which included the CCAT-PF. Patients and caregivers completed the CCAT-PF independently from each other as well as the Depression, Anxiety, Stress Scales (DASS-21) [27] (patients only), attitudes to ACP [25], and the Patient Decision Making Scale (PDMS) [25]. On conclusion of the RCT, sample size calculations indicated that additional CCAT-PF data was required for sufficient reliability. Additional patients were recruited, with completion of the questionnaire implying consent. Ethical approval was granted by the institution’s Health Research Ethics Committee: RES-20–0000-112C.

### Measurements

#### ***Cancer Communication Assessment Tool for Patients and Families (CCAT-PF) ***[17]

The CCAT-PF (Appendix 1) was developed to measure congruence in patient-caregiver family communication, with the potential for it to be used as a clinical screening tool to assess the level of family risk for communication. The CCAT-PF has analogous patient (CCAT-P) and family (CCAT-F) instruments, which consist of 18 items within eight domains: general communication and interaction style, reluctance to report side effects, treatment and care goals, trade-off between side effects and quality of life, family support of decisions, patient and family perspectives about physicians’ decisions and communication, family communication (five items), and hospice care (one item). Responses for each item are reported on a 6-point Likert scale (1 = strongly agree/all the time, 6 = strongly disagree/never). Scoring instructions for the CCAT-P, CCAT-F, and CCAT-PF have been published elsewhere [17, 28]. The range of scores for the CCAT-P/CCAT-F is 18–108 and for the CCAT-PF is 0–90. Higher scores indicate greater conflict and therefore poorer concordance in communication.

#### ***Depression, Anxiety, and Stress Scale (DASS-21) ***[27]

The DASS-21 is used to observe negative emotional reactions (depression, anxiety, and stress). It has acceptable internal reliability for its depression subscale (*α* = 0.90) and anxiety subscale (*α* = 0.70), with concurrent validity to measures of suicidal ideation, quality of life, self-rated health, and depressed mood.

### *Attitudes towards ACP *[25]

This is a non-validated scale developed by the research team and used in a previous feasibility study and the RCT intervention in patients and caregivers [21, 25]. It measures understanding of ACP, satisfaction, and distress experienced in undertaking ACP on a Likert scale from 0 to 10.

### *Preparation for Decision Making Scale (PDMS) *[25]

The PDMS was used in the RCT intervention in patients and caregivers [21]. It assesses a participant’s perception of how useful a decision support intervention is in preparing them for making a health decision and communicating with their practitioner at a consultation, visit, and making a health decision. With strong internal consistency (*α* = 0.92–0.96), it discriminates significantly between patients who do and do not find a decision support intervention helpful.

### Statistical analysis

Incomplete pairs of CCAT-PF questionnaires whereby > 40% of the CCAT-P and/or CCAT-F items were missing were excluded from the analysis [19]. Individual CCAT-P, CCAT-F, and CCAT-PF scores were calculated [17, 28] and presented as mean (*SD*) and the absolute difference between dyads. Allowing for data skewness, Spearman’s rho was used to examine the correlation between two scales and final CCAT-P and CCAT-F scores, whilst weighted kappa was used to examine the agreement between each item.

Bartlett’s test of sphericity was used to assess the relationships between items and their suitability for the factor analysis, and the Kaiser–Meyer–Olkin criteria was used for sampling adequacy and to assess the strength of the relationships among the variables. Exploratory principal component factor analysis was undertaken to identify specific domains of this scale. Both scree plot and Kaiser-Guttman criteria were used to determine the number of factors to be included. Pearson’s correlation coefficient was used to assess correlations between various domains of CCAT, factors and DASS domains, PDMS total score, and Cronbach’s *α* to assess the internal validity. All analyses were performed using Stata16 (StataCorp LLC, College Station, TX, USA), and level of significance was set at *p* < 0.005.

## Results

### Study participants

**Fig. 1:**
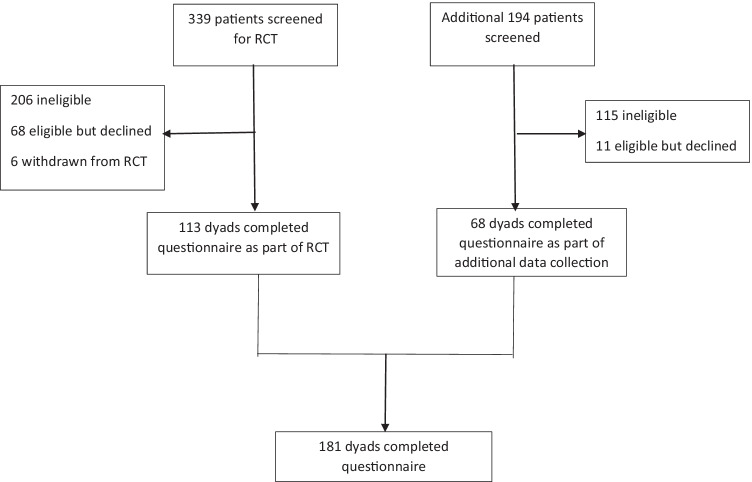
Participant enrollment

From a total of 533 patients screened, 212 met the eligibility criteria, and data from 181 dyads were analysed (85.4% response rate) (Fig. 1). Cohort descriptions, including demographic and clinical data, are summarised in Table 1. The mean patient age was 69.1 (*SD* 12.9) and caregiver age 60.1 (*SD* 14.4). The majority of participants were female (patients 60.2%, caregivers 61.3%) and were married or in de-facto relationships (patients 68%, caregivers 81.8%). Most caregivers were spouses or partners of patients (59.1%), with close to a third being children or in-laws. Gastrointestinal cancer was the most common diagnosis (28.9%), followed by lung cancer (18.9%), with most having lived with their diagnosis for 1–5 years (36.3%).

**Table 1:** Participant demographics

	Patient	Caregiver
Age, mean (*SD*)	69.1 (12.9)	60.1 (14.4)
Sex Male Female	72 (39.8)	
		70 (38.7)
	109 (60.2)	111 (61.3)
Marital status		
Married/defacto	125 (68.0)	152 (81.8)
Widowed	24 (13.3)	3 (1.7)
Single	14 (7.7)	20 (11.1)
Separated/divorced	18 (9.9)	6 (3.3)
County of birth		
Australia/New Zealand	124 (68.5)	137 (76.1)
Europe	37 (20.4)	24 (13.3)
Other Asia Pacific	9 (5.0)	7 (3.9)
North/South America	5 (2.8)	5 (2.8)
Other	6 (3.3)	7 (3.9)
Relationship to caregiver		
Spouse/partner	107 (59.1)	
Parent/parent-in-law	49 (27.1)	
Sibling	8 (4.4)	
Child	8 (4.4)	
Friend	6 (3.3)	
Cancer diagnosis		
Gastrointestinal	52 (28.9)	
Lung	34 (18.9)	
Genitourinary	27 (15.0)	
Breast	26 (14.4)	
Gynaecological	15 (8.3)	
Haematological	11 (6.1)	
Skin	6 (3.3)	
Other	9 (5.0)	
Time since diagnosis		
< 6 moths	42 (23.5)	
6–12 months	29 (16.2)	
1–5 years	65 (36.3)	
> 5 years	43 (24.0)	

### Descriptive statistics and internal consistency of CCAT

The mean CCAT-P score was 46.2 (*SD* 9.8), and CCAT-F score was 45.7 (*SD* 9.4). The mean CCAT-PF score was 24.1 (*SD* 8.0). Dyads showed the greatest absolute differences for the following items: if treatment caused financial hardship for my family, I would not take it (2.1); I am willing to take treatment that causes me a significant amount of pain if I can live a few months longer (1.9); in general, side effects are not really important when I consider my larger goals of treatment (1.8); if treatment made me sick every day, I would not take it (1.6); my family’s acceptance of my treatment decisions depends on how much they like my doctor(s) (1.6). At the same time, 82.6% of patient-carers pairs provided an identical response to Q17 (My family blames my cancer on me not having taken better care of myself), and thus, this item was removed from the factor analysis due to its severe skewness (Sk = 2.7, kurtosis = 10.0).

The results reported in Table 2 show poor concordance between patient and caregiver items. There was minimal to no agreement between CCAT-P and CCAT-F (weighted kappa ranges from 0.01 to 0.31), with the strongest agreement attributed to questions 16 and 4: (frustration with family due to overprotection and willingness to undertake any treatment, hopeful that medical science will find a cure). The internal consistency (Cronbach’s alpha) was 0.58 for CCAT-P, 0.61 for CCAT-F, and 0.49 for CCAT-PF.

**Table 2:** Concordance of cancer communication assessment between patients and their family caregivers

CCAT items	Patient response	Caregiver response	Absolute difference between patient and caregiver^*^	Correlation between dyads	
	Mean (*SD*)	Mean (*SD*)	Mean (*SD*)	Weighted *κ*	*p-*value
1 My family plays a big role in the decisions I make about my cancer treatment	2.25 (1.64)	2.42 (1.62)	1.48 (1.58)	0.13	0.009
2 I hesitate to mention treatment side effects to my doctors or nurses	5.28 (1.34)	4.75 (1.44)	1.37 (1.47)	0.04	0.213
3 In general, side effects are not really important when I consider my larger goals of treatment	3.08 (1.75)	3.89 (1.61)	1.77 (1.36)	0.14	0.002
4 Medical science may find a cure for cancer so I am willing to take any treatment now to stay alive	2.53 (1.79)	2.69 (1.71)	1.37 (1.39)	0.28	< 0.001
5 If treatment caused financial hardship for my family, I would not take it	3.61 (1.94)	5.25 (1.35)	2.09 (1.83)	0.08	0.018
6 My family and I have different views about the goal of treatment	5.29 (1.34)	5.4 (1.11)	0.92 (1.36)	0.13	0.007
7 If treatment made me sick every day, I would not take it	3.08 (1.81)	3.04 (1.56)	1.59 (1.44)	0.17	< 0.001
8 I could see that there could come a point when taking treatment would not be worth the discomfort it causes	2.26 (1.52)	2.13 (1.31)	1.22 (1.34)	0.16	< 0.001
9 I am willing to take treatment that causes me a significant amount of pain if I can live a few months longer	3.47 (1.92)	4.4 (1.69)	1.93 (1.61)	0.12	0.008
10 I value my family’s judgement about treatment decisions	1.99 (1.37)	1.98 (1.02)	1.08 (1.15)	0.10	0.017
11 My family’s acceptance of my treatment decisions depends on how much they like my doctor(s)	4.44 (1.90)	4.57 (1.69)	1.57 (1.67)	0.17	< 0.001
12 It is important to base decisions about my cancer treatment on sources of information other than my doctor	4.46 (1.61)	4.40 (1.37)	1.54 (1.34)	0.04	0.196
13 My family does not really listen when I talk about my cancer	5.32 (1.31)	5.55 (1.09)	0.90 (1.37)	0.01	0.391
14 I avoid talking about cancer to my family because I don’t want to upset them	4.37 (1.53)	4.93 (1.39)	1.42 (1.35)	0.13	0.003
15 I don’t tell my family about my problems because there is nothing they can do to help	4.58 (1.47)	5.22 (1.06)	1.23 (1.24)	0.13	0.003
16 I am frustrated when my family is overprotective of me because of my cancer	4.87 (1.50)	4.83 (1.36)	1.01 (1.12)	0.31	< 0.001
17 My family blames my cancer on my not having taken better care of myself	5.84 (0.60)	5.76 (0.79)	0.31 (0.78)	0.17	0.002
18 I would feel uncomfortable if the doctor began to talk to me about hospice care	4.72 (1.69)	4.91( 1.50)	1.45 (1.53)	0.10	0.040
	46.15 (9.76)	45.73 (9.39)	24.07 (7.97)	0.19^**^	0.010

### Item analysis and evaluation of the scale structure

As per the study by Haun et al. [19], we assumed different subdomains of familial cancer-related communication and thus completed a factor analysis on the discrepancy scores between CCAT-P and CCAT-F. The result of Bartlett’s test showed a satisfactory inter-correlation between items (*χ*^2^ (136) = 259.1, *p* < 0.001, after excluding question 17), suggesting a data set adequacy for the factor analysis and the Kaiser–Meyer–Olkin measure of sampling adequacy was low (*KMO* = 0.56) but satisfactory for the factor analysis.

A 3-factor or 4-factor solution was suggested following examination of the Scree plot and Kaiser-Gutmann analysis. Factor 1 and Factor 3 were identical in both solutions. However, Factor 4 in the four-factor solution showed low internal consistency (*α* = 0.22) thus making us opt for a 3-factor solution. The eigenvalues for these subscales were all > 1 and were as follows: Factor 1 2.24, accounting for 13.2% of the variance; Factor 2 1.83, accounting for an additional 10.8% of the variance; and Factor 3 1.44 accounting for an additional 8.5% of variance. The final three subscales corresponded with 32.5% of the total variance, with factor loadings of > 0.40 throughout (Table 3).

**Table 3:** Scale characteristics for CCAT items

CCAT items	Factor 1	Factor 2	Factor 3
Scale 1 Disclosure			
15 I don’t tell my family about my problems because there is nothing they can do to help	**0.65**	0.13	0.03
10 I value my family’s judgement about treatment decisions	**0.59**	− 0.26	− 0.30
13 My family does not really listen when I talk about my cancer	**0.57**	− 0.00	0.20
14 I avoid talking about cancer to my family because I don’t want to upset them	**0.57**	0.11	0.01
Scale 2 Limitation of treatment			
8 I could see that there could come a point when taking treatment would not be worth the discomfort it causes	0.01	0.69	− 0.00
18 I would feel uncomfortable if the doctor began to talk to me about hospice care	− 0.08	0.67	0.08
16 I am frustrated when my family is overprotective of me because of my cancer	0.12	0.51	0.22
7 If treatment made me sick every day, I would not take it	0.29	0.49	− 0.16
Scale 3 Treatment decision making			
6 My family and I have different views about the goal of treatment	0.20	− 0.07	0.66
12 It is important to base decisions about my cancer treatment on sources of information other than my doctor	0.24	0.25	0.58
11 My family’s acceptance of my treatment decisions depends on how much they like my doctor(s)	0.22	− 0.09	0.57
9 I am willing to take treatment that causes me a significant amount of pain if I can live a few months longer	− 0.27	0.13	0.52
Items without clear factor loadings			
1 My family plays a big role in the decisions I make about my cancer treatment	0.33	− 0.07	0.04
2 I hesitate to mention treatment side effects to my doctors or nurses	0.36	0.07	0.15
3 In general, side effects are not really important when I consider my larger goals of treatment	− 0.18	0.17	0.17
4 Medical science may find a cure for cancer so I am willing to take any treatment now to stay alive	0.17	− 0.27	0.05
5 If treatment caused financial hardship for my family, I would not take it	− 0.05	− 0.22	0.24
Items excluded due to severe skewness			
17 My family blames my cancer on my not having taken better care of myself			
Eigen value	2.24	1.83	1.44
Cronbach’s *α*	0.53	0.53	0.43
Explained variance	13.2%	10.8%	8.5%
Total variance	32.5%		

The pattern matrix in Table 3 revealed Factor 1 to consist of four items. This factor was labelled ‘disclosure’ and demonstrated moderate internal consistency. The second and third factor consisted of 4 items each, relating to treatment decisions and were labelled ‘limitation of treatment’ and ‘treatment decision making’ respectively, both with moderate internal consistency. Five items were excluded due to insufficient factor loading.

### Assessment of reliability and validity

Table 3 shows the Cronbach’s *α* of the 3 identified factors: disclosure, *α* = 0.52; limitation of treatment, *α* = 0.52; and treatment decision making, *α* = 0.43. Regarding concurrent validity (Table 4), CCAT-P and CCAT-F both had strong Pearson correlations with corresponding decision-making (PDMS) scores for patients (*r* = 0.92, *p* < 0.001) and caregivers (*r* = 0.93, *p* < 0.001) respectively. At the emotional level, there was no significant correlation found between the DASS-21, CCAT-P, or CCAT-PF for any of the 3 factors. However, in exploring correlations between patient/caregiver communication and attitudes to ACP, the CCAT-P score showed a weak positive correlation with ‘level of confidence in discussion of possible future health care needs/wishes with family members/friends’ (*r* = 0.21, *p* = 0.029) and the ‘benefits of considering an ACP’ (*r* = 0.19, *p* = 0.044). Likewise, a weak positive correlation was found between the CCAT-F and ‘distress caused by discussing my family member’s/friend’s possible future care health needs/wishes with him/her’ (*r* = 0.216, p = 0.023) and level of confidence in discussion of possible future health care needs/wishes with health professionals (*r* = 0.214, *p* = 0.025). There were no significant correlations between CCAT-PF and patient/caregiver attitudes towards ACP.

**Table 4:** Concurrent validity of Cancer Communication Assessment Tool

	Patients		Caregivers	
	CCAT-P	CCAT-PF	CCAT-F	CCAT-PF
Preparation for decision making	0.915^*^	− 0.07	0.929^*^	− 0.07
DASS-21^#^			N/A	N/A
Depression	− 0.137	0.066		
Anxiety	− 0.085	0.135		
Stress	− 0.097	0.046		
Attitudes to advance care planning				
Rating my current understanding of ACP	− 0.188	− 0.076	0.094	0.095
Satisfaction with opportunity to consider my/my family member or friends possible future health care needs and wishes with health care professionals	− 0.033	− 0.046	0.112	0.015
Distress caused by thinking about my/my family member or friends possible future health care needs and wishes if I became unwell	0.025	− 0.17	0.147	0.052
Distress caused by discussing my/my family member or friends possible future care health needs and wishes with others/him or her	0.092	− 0.064	0.216^**^	− 0.009
Importance of making and informing others about decisions related to my/my family members’ or friend’s possible future health care needs and wishes	0.04	0.0002	0.159	0.017
Level of confidence in discussion of my/my family members’ or friend’s possible future health care needs and wishes with health professionals	0.169	0.0308	0.214^**^	0.133
Level of confidence in discussion of my possible future health care needs and wishes with family members/friends^#^	0.209^**^	− 0.147 Abstract_Sec Abstract_Sec	N/A	N/A
Benefit of considering my advanced care plan	0.192^**^	− 0.155	0.183	− 0.015
Importance of considering advance care planning when living with a cancer diagnosis is^#^	0.03	0.003	N/A	N/A

^*^(*p* < 0.001)

^**^p < 0.05

^#^Questions completed in the RCT by patients only

## Discussion and conclusion

Our study extends the understanding of potential areas of discordant communication between cancer patients and their caregivers, reporting a mean CCAT-PF score of 24.07, which was marginally lower to that reported in the original American study (25.9) [17] and comparable to that reported in a Korean study (23.7) [18]. We confirm the overall disagreement between patient and caregiver reporting of items in the CCAT-PF and reinforce findings that demonstrated the multidimensional nature of dyadic communication across relatively distinct themes. Finally, we describe a three-factor model for the CCAT-PF, achieving sufficient moderate consistency.

A high number of psychosocial variables such as socio-economic variables, ethnicity, and relationship quality are known to affect dyadic concordance in cancer communication [20, 29, 30]. Thus, it is unsurprising that low kappa values (< 0.2) were shown in the level of agreement between patient and caregiver reports for the majority of items, indicating disagreement between patient and caregiver in rating each item. Our findings reflect that of Siminoff et al. [17] and Shin et al. [18], with the overall disagreement similar to that reported in the Korean cohort (Spearman’s rho 0.18 vs. 0.19) [18]. This confirms the appropriateness of the scale as a measure of discordance [17] and supports the view that overall, families avoid communication around distressing topics [31], regardless of the influence of culture and ethnicity on communication styles and discordance [20, 32].

Four of the five items with the highest absolute difference between CCAT-P and CCAT-F were similar to that found in the Korean cohort [18]. Items of discordance in both studies related to consideration of treatment decisions in financial hardship, prolonging survival despite pain, and tolerating treatment side effects in the context of broader treatment goals and partiality towards the treating doctor. These findings are in keeping with the known direct and indirect financial impact of cancer treatment which attribute to significant family stress [33] and the overestimation of cancer pain and other symptoms by caregivers [34]. Thus, a patient may choose to avoid treatment that may impose financial inconvenience or burden on family, and likewise, a caregiver may find it hard to witness a patient endure suffering with treatment that may cause significant symptoms, despite the opportunity for life prolongation.

High CCAT-P and CCAT-F scores were weakly associated with patient/caregiver confidence and distress related to having difficult conversations on future care planning and strongly associated with patient and caregiver preparation for decision-making. Patients and their loved ones can avoid discussion of prognosis [35] and lack the confidence to initiate dialogue with health practitioners [36] and hence stay away from conversations about end-of-life decisions for fear that these might provoke distress [37]. This general protectiveness about avoiding distress [38] may block important discussions and limit openness to ACP. Despite this, and unlike the findings from the original study, we were unable to show a significant correlation between the CCAT-PF and patient depression and quality of life domain scores. This may suggest that in the Australian setting, it may not be presence of mood disorders such as depression and quality of life issues as such that contributes to discordance in communication, but more so a possible avoidance of conversations that are perceived to potentially cause distress [39]. Our factor analysis demonstrated a 3-factor structure for 12 of the 18 original items, based on a principal components factor analysis scree plot and Kaiser-Guttman criteria. We partially reproduced, with some variations in items, the disclosure and limitation of treatment scales and achieved a reasonably similar internal consistency in the disclosure scale as demonstrated by Haun et al. (*α* = 0.66 vs. *α* = 0.53) [19], and allowing for the diversity of themes being assessed, the overall reliability of the subscales is reasonable. Strong concurrent validity is demonstrated with a measure of decision-making. Our cohort representation of autonomous and reasonably well-educated patients with financial means may explain why some of the items in the original scale (1–5) may have resonated less with this study population and not loaded well onto factors evident here.

This study has several limitations. Firstly it was conducted in a single location, with a relatively affluent population with high literacy thus affecting generalizability within the broader Australian setting. Secondly, this was a sub study of a larger RCT [21, 22], and we were limited in the assessment of validity with more specific instruments than if we had planned this as a primary validation study. Finally, we did not follow up the CCAT longitudinally, which may have demonstrated more specifics of communication patterns over time in a cancer population.

## Conclusion

There has been a dearth of measures of the concordance of communication between patient and caregiver, yet such communication is critical to care planning and decision-making at the end-of-life. Our preliminary validation of the CCAT-PF in the Australian setting has demonstrated some similar psychometric properties as in previously published studies. We perceive that this early Australian-based refinement of the CCAT offers an opportunity for its further refinement to confirm its utility as a reliable tool to assess dyadic communication and understand its impact. It paves the way for further research on how we evaluate communication in the clinical setting and improve outcomes for families as a unit of care.

## Supplementary Information

Below is the link to the electronic supplementary material.

Supplementary file1 (PDF 454 KB)

## Data Availability

Cabrini Palliative Care Research Department retains primary control of the data presented in this manuscript. Data may be made available for external review if permission is obtained.
