# Supplementary material for: Patient-caregiver communication concordance in cancer—refinement of the Cancer Communication Assessment Tool in an Australian sample
Source: Support Care Cancer. 2022 May 25;30(9):7387–96. doi: 10.1007/s00520-022-07163-7 (PMC9385757; doi:10.1007/s00520-022-07163-7)
Supplement: Supplementary file 1 — Supplementary file1 (PDF 454 KB) [file 520_2022_7163_MOESM1_ESM.pdf]

Cancer Communication Assessment Tool - Patient Version

	**All of the time**	**Most of the time**	**Half of the time**	**Some of the time**	**A little of the time**	**Never**
1. My family plays a big role in the decisions I make about my cancer treatment.	1	2	3	4	5	6
2. I hesitate to mention treatment side effects to my doctors or nurses.	1	2	3	4	5	6
	**Strongly Agree**	**Mostly Agree**	**Slightly Agree**	**Slightly Disagree**	**Mostly Disagree**	**Strongly Disagree**
3. In general, side effects are not really important when I consider my larger goals of treatment.	1	2	3	4	5	6
4. Medical science may find a cure so I am willing to take any treatment now to stay alive.	1	2	3	4	5	6
5. If treatment caused financial hardship for my family, I would not take it.	1	2	3	4	5	6
	**All of the time**	**Most of the time**	**Half of the time**	**Some of the time**	**A little of the time**	**Never**
6. My family and I have different views about the goal of treatment.	1	2	3	4	5	6
	**Strongly Agree**	**Mostly Agree**	**Slightly Agree**	**Slightly Disagree**	**Mostly Disagree**	**Strongly Disagree**
7. If treatment made me sick every day I would not take it.	1	2	3	4	5	6
8. I could see that there could come a point when taking treatment would not be worth the discomfort it causes.	1	2	3	4	5	6
9. I am willing to take treatment that causes me a significant amount of pain if I can live a few months longer.	1	2	3	4	5	6
	**All of the time**	**Most of the time**	**Half of the time**	**Some of the time**	**A little of the time**	**Never**
10. I value my family's judgment about treatment decisions.	1	2	3	4	5	6
	**Strongly Agree**	**Mostly Agree**	**Slightly Agree**	**Slightly Disagree**	**Mostly Disagree**	**Strongly Disagree**
11. My family's acceptance of my treatment decisions depends on how much they like my doctor(s).	1	2	3	4	5	6

	All of the time	Most of the time	Half of the time	Some of the time	A little of the time	Never
12. It is important to base decisions about my cancer treatment on sources of information other than my doctor.	1	2	3	4	5	6
13. My family does not really listen when I talk about my cancer.	1	2	3	4	5	6
14. I avoid talking about cancer to my family because I don't want to upset them.	1	2	3	4	5	6
15. I don't tell my family about my problems because there is nothing they can do to help.	1	2	3	4	5	6
16. I am frustrated when my family is overprotective of me because of my cancer.	1	2	3	4	5	6
17. My family blames my cancer on my not having taken better care of myself.	1	2	3	4	5	6
	**Strongly Agree**	**Mostly Agree**	**Slightly Agree**	**Slightly Disagree**	**Mostly Disagree**	**Strongly Disagree**
18. I would feel uncomfortable if the doctor began to talk to me about palliative/hospice care.	1	2	3	4	5	6

Cancer Communication Assessment Tool - Caregiver Version

	**All of the time**	**Most of the time**	**Half of the time**	**Some of the time**	**A little of the time**	**Never**
19. Our family plays a big role in making decisions about cancer treatment.	1	2	3	4	5	6
20. My family member/friend hesitates to mention treatment side effects to doctors or nurses.	1	2	3	4	5	6
	**Strongly Agree**	**Mostly Agree**	**Slightly Agree**	**Slightly Disagree**	**Mostly Disagree**	**Strongly Disagree**
21. In general, side effects are not really important when I consider the larger goals of my family member's/friend's treatment.	1	2	3	4	5	6
22. Medical science may find a cure for cancer so I want my family member/friend to take any treatment now to stay alive.	1	2	3	4	5	6
23. If my family member's/friend's treatment caused financial hardship, I would not want him/her to take it.	1	2	3	4	5	6
	**All of the time**	**Most of the time**	**Half of the time**	**Some of the time**	**A little of the time**	**Never**
24. My family member/friend and I have different views about the goal of treatment.	1	2	3	4	5	6
	**Strongly Agree**	**Mostly Agree**	**Slightly Agree**	**Slightly Disagree**	**Mostly Disagree**	**Strongly Disagree**
25. If treatment made him/her sick every day, I would not want him/her to take it.	1	2	3	4	5	6
26. I could see that there could come a point when taking treatment would not be worth the discomfort it causes.	1	2	3	4	5	6
27. My family member/friend should be willing to take treatment that causes him/her a significant amount of pain if he/she will live a few months longer.	1	2	3	4	5	6
	**All of the time**	**Most of the time**	**Half of the time**	**Some of the time**	**A little of the time**	**Never**
28. My family member/friend values my judgment about treatment decisions.	1	2	3	4	5	6
	**Strongly Agree**	**Mostly Agree**	**Slightly Agree**	**Slightly Disagree**	**Mostly Disagree**	**Strongly Disagree**
29. My acceptance of treatment decisions depends on how much I like the doctor(s).	1	2	3	4	5	6

	All of the time	Most of the time	Half of the time	Some of the time	A little of the time	Never
30. It is important to base decisions about cancer treatment on sources of information other than the doctor.	1	2	3	4	5	6
31. I can't really listen when my family member/friend talks about his/her cancer.	1	2	3	4	5	6
32. I avoid talking about cancer to my family member/friend because I don't want to upset him/her.	1	2	3	4	5	6
33. My family member/friend does not tell me about his/her problems because he/she thinks there is nothing I can do to help.	1	2	3	4	5	6
34. My family member/friend is frustrated when I am overprotective because of his/her cancer.	1	2	3	4	5	6
35. I blame cancer on my family member/friend not having taken better care of him/herself.	1	2	3	4	5	6
	**Strongly Agree**	**Mostly Agree**	**Slightly Agree**	**Slightly Disagree**	**Mostly Disagree**	**Strongly Disagree**
36. I would feel uncomfortable if the doctor began to talk to my family member/friend about palliative/hospice care.	1	2	3	4	5	6
